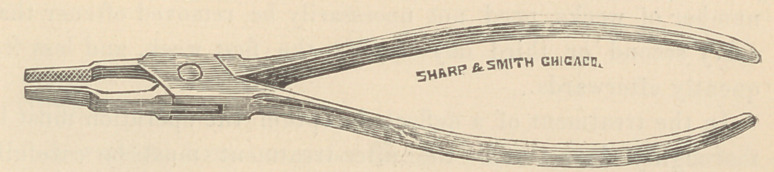# Case of Deflection of the Septum Narium

**Published:** 1883-08

**Authors:** E. Fletcher Ingals

**Affiliations:** Professor of Laryngology, Rush Medical College; Professor Diseases of Throat and Chest Woman’s Medical College, etc., Chicago


### ARTICLE III.

### CASE OF DEFLECTION OF THE SEPTUM NARIUM. By E. FLETCHER INGALS, A.M., M D.,

Professor of Laryngology, Rush Medical College; Professor Diseases of Throat and Chest Woman's Medical College, etc., Chicago.

The little patient, F. McG., aged 28 months, was brought to me by the family physician, Dr. P. J. Rowan, of this city, about the middle of last February, on account of obstructed nasal respiration and deformity of the nose due to deflection of the septum.

The child was apparently perfectly healthy excepting a rhinitis. The mother told me that several weeks previously he had received two or three severe falls, striking upon his nose, and since that time she had noticed some deformity and gradually increasing difficulty in nasal respiration, with resulting restlessness at night.

I found the end of the nose bent to the right and flattened, and the septum bent in the opposite direction so as to completely occlude the left naris. A few days later, with the assistance of Dr. Rowan, I gave the child chloroform, and, with a nasal forceps, bent the septum forcibly back to its normal position, where it was retained by means of a plug of gutta-percha inserted into the left naris, and held in position by its end being lodged in the pocket at the tip of the nose.

A couple of days later this plug was replaced by a gutta-percha tube accurately fitted to the nasal cavity, which was worn for the next five weeks until a perfect cure was effected.

The forceps which I use for the purpose is similar to that of Dr. William Adams, of London, England. It has long, flat blades which when closed do not touch each other by about two millimeters, thus leaving a space which prevents too severe contusion of the tissues of the septum. Between the ends of the blades and the joints there is a much wider space as represented in the annexed cut, which allows for the thicker tissues between the nostrils.

In performing this operation it is important to bend the septum thoroughly or even to break it so as to destroy its resiliency, otherwise it will immediately spring back to its abnormal position, and even though the plug be worn for weeks, a cure will not be effected.

In deflection of the septum, as I have formerly pointed out,* there are various degrees of deformity due either to simple fracture or distortion of the septum, or to enlargement of its cartilaginous portion with consequent bending. The latter cases cannot be cured by the method here described, but, as stated in the article already referred to, the excess of tissue must be removed.

After the septum has been replaced it may be held in position

---

*Archives of Laryngology*, October, 1882; reprinted in the CHICAGO MEDICAL JOURNAL AND EXAMINER, December, 1882.

9

by plugs of cotton, or sponge, or by clamps or solid plugs, fitted for the purpose. The porous substances are objectionable because they soon become very offensive and have to be removed, which causes the patient much pain.

The clamps, as recommended by Adams, are theoretically valuable but practically undesirable.

A properly-fitted smooth plug or tube, which will not absorb moisture, will be found most satisfactory. I have found gutta-percha to be the best substance of which to make the plug or tube, as it can be easily and accurately molded by the surgeon to fit the case in hand; while hard-rubber tubes or plugs must be made by the instrument-maker, and can seldom be made to fit. It is not difficult to construct a tube out of gutta-percha by molding it over a small wooden plug shaped tc correspond to the nasal cavity.

After the operation the plug, which will have to be worn for a number of weeks, need not necessarily be removed oftener than every second or third day during the first week, and less frequently afterwards.

In the treatment of a deflected septum the operation must be thoroughly done, and the after-treatment must be carefully attended to; thus we may relieve our patients of the manifold evils which attend occlusion of the nares, and, in a majority of cases, we may correct the distortion of the nose which results from bending of its supports.

## Figures and Tables

**Figure f1:**